# Induction of Myogenic Differentiation Improves Chemosensitivity of Chemoresistant Cells in Soft-Tissue Sarcoma Cell Lines

**DOI:** 10.1155/2020/8647981

**Published:** 2020-03-26

**Authors:** Lucy E. Dawson, Luca D'Agostino, Abraham A. Hakim, Richard D. Lackman, Spencer A. Brown, Richard B. Sensenig, Zeus A. Antonello, Igor I. Kuzin

**Affiliations:** ^1^Cooper University Hospital, Camden, NJ, USA; ^2^Cooper Medical School of Rowan University, Camden, NJ, USA; ^3^MD Anderson Cancer Center at Cooper, Camden, NJ, USA

## Abstract

Rhabdomyosarcoma (RMS) and rhabdoid tumors (RT) are rare soft-tissue malignancies with the highest incidence in infants, children, and adolescents. Advanced, recurrent, and/or metastatic RMS and RT exhibit poor response to treatment. One of the main mechanisms behind resistance to treatment is believed to be intratumoral heterogeneity. In this study, we investigated the myogenic determination factor 1 (MYOD1) and Noggin (NOG) markers in an embryonal RMS (ERMS) cell line and an RT cell line and the differential response of the MYOD1 and NOG expressing subpopulations to chemotherapy. Importantly, we found that these markers together identify a subpopulation of cells (MYOD1+ NOG+ cells) with primary resistance to Vincristine and Doxorubicin, two commonly used chemotherapies for ERMS and RT. The chemoresistant MYOD1+ NOG+ cells express markers of undifferentiated cells such as myogenin and ID1. Combination of Vincristine with TPA/GSK126, a drug combination shown to induce differentiation of RMS cell lines, is able to partially overcome MYOD1/NOG cells chemoresistance.

## 1. Introduction

Rhabdomyosarcoma (RMS) and rhabdoid tumors (RT) are rare soft-tissue malignancies with the highest incidence in infants, children, and adolescents. About 400 to 500 new cases of RMS and only about 15 new cases of RT occur each year in the United States, comprising approximately 3% of all childhood cancers. Although RMS rarely occurs in adults, the outcomes are significantly worse [1]. Many adult patients with advanced RMS die because their cancer exhibits or develops resistance to available therapies. RMS is comprised of two main histological variants, alveolar and embryonal (ERMS). ERMS has a more complex and heterogeneous genetic profile but has an overall better outcome, as high as 90% 5-year survival for the low-risk group [2]. However, when ERMSs are advanced, recurrent, and/or metastatic, they are classified as high risk and exhibit poor response to treatment (chemoresistance), having a progression-free survival less than 1.5 years with a 5-year survival rate as low as 20% [3–5]. In both children and adults, RMS and RT are treated with a combination of therapies including surgery, radiation, and chemotherapy [6, 7].

One of the main mechanisms behind resistance to treatment and recurrence is believed to be intratumoral heterogeneity. Heterogeneity in genomic, transcriptomic, and proteomic profiles among the cells constituting the tumor manifests as a differential response to the applied therapies [8–10]. Although clinical tumors may respond by regressing in size or even becoming undetectable upon treatment, therapeutic intervention may facilitate the expansion of an initially small population of nonresponsive cells and reconstitute the primary tumor and/or metastasize [11]. Intratumoral heterogeneity represents therefore a major obstacle to effective cancer treatment [12]. Both main variants of RMS and RT have been reported to have intratumoral heterogeneity in patients [13, 14]. In embryonal rhabdomyosarcoma, intratumor diversity has been correlated with reduced survival [15] and it has been shown to change under treatment in patients [16, 17].

In order to devise therapeutic approaches able to target a heterogeneous tumor population, it is therefore important not only to characterize the different tumor subpopulations but also to understand how cell subpopulations may change upon treatment. Such information can guide the design of high-order combined therapies [11]; however, only limited data exist regarding RMS and RT intratumor heterogeneity changes under treatment.

In this study, the differential response to chemotherapy associated with the heterogeneity of myogenic determination factor 1 (MYOD1) and Noggin (NOG) markers in ERMS and RT cell lines was investigated. The RD cell line, one of the most commonly used for RMS investigations [18], was examined as well as the A-204 cell line, originally identified as RMS but later classified as a rhabdoid tumor (RT) [19]. RMS tumors have been reported to be positive for MYOD1 with marked heterogeneity between cells [18], while RT are believed to be negative for MYOD1 [20, 21]. MYOD1 is one of the four myogenic regulatory genes that drive differentiation of muscle cell precursors to mature muscle cells, and it has been shown to be sufficient to convert nonmuscle cells into myoblast-like cells [22]. Myogenic transcription factors such as MYOD1 are normally tightly regulated during homeostasis and tissue repair [22, 23], but in RMS, MYOD1 is deregulated or mutated, resulting in reduced survival of the patients [15, 24]. NOG is another tightly regulated protein required for correct muscle morphogenesis [25] and adult muscle homeostasis [26] and repair [27]. NOG antagonizes bone morphogenetic proteins (BMPs) which, by binding to BMP-receptors, modulate proliferation and differentiation. Inhibitors of differentiation (Id) proteins are important downstream effectors of BMP signaling and are deregulated in several cancers [28]. In myoblasts, Id proteins inhibit cell differentiation and potentiate cell proliferation by sequestering and antagonizing MYOD1 and myogenin transcription activity [29]. In RMS, morphogenetic signaling is aberrant leading to altered proliferation and differentiation of myogenic precursors and/or differentiated cells [30]. Further analysis of the myogenic determinant MYOD1 and the morphogenetic protein NOG could provide insights into the mechanisms of chemoresistance, possibly leading to improved treatments and clinical prognoses.

## 2. Materials and Methods

### 2.1. Cell Lines

RD (ATCC® CCL-136™) and A-204 (ATCC® HTB-82™) were purchased from American Type Culture Collection (Manassas, VA) and cultured under standard conditions (37°C, 5% CO_2_) in DMEM High Glucose supplemented with 10% fetal bovine serum. Cells were seeded 24 hrs prior to each assay as indicated below.

### 2.2. Drugs Preparation and Treatments

All drugs were prepared and stored as per manufacturer indications. In detail, Doxorubicin (Sigma Aldrich, St. Louis, MO), 12-O-tetradecanoylphorbol-13-acetate (TPA) (EMD Millipore, Temecula, CA), and GSK126 (AdooQ Bioscience, Irvine, CA) were resuspended in 100% DMSO and stored at −20°C. Vincristine (AdipoGen, San Diego, CA) was resuspended in sterile water and stored at 4°C. Cells were treated after 24 hours from seeding in 6-well plates at the doses indicated in each figure. Drugs were diluted in full media and final DMSO concentration on cells never exceeded 0.01%.

### 2.3. Flow Cytometry

Intracellular staining was performed on fixed and permeabilized RMS and RT cell suspensions with Transcription Factor Buffer Set (BD Biosciences, Jose, CA) prior to staining with fluorochrome-conjugated anti-human antibodies following manufacturer's protocol with minor modifications. Briefly, cells were incubated in Fix/Perm buffer for 40 min, washed with Perm/Wash buffer, and incubated for 15 min with 2% rabbit/2% mouse normal sera to block nonspecific binding. Cells were then stained for 2 hrs with mixture of fluorochrome-conjugated antibodies in Perm/Wash buffer: Bcl-2 (clone 100) from BioLegend, MYOD1 (polyclonal) and NOG (polyclonal) from Bioss Antibodies, MYOD1 (clone SPM427) and myogenin (clone MGN185 + F5D) from Novus Biologicals, and ID1 (clone B-8) from Santa Cruz Biotechnology, followed by staining with PE-streptavidin (Invitrogen) if biotinylated antibody was included in the staining mixture. The optimal antibody dilution was established by titration curve. Matching isotype controls were prepared as per standard flow cytometry protocols to determine background signals. All steps were performed on ice and samples kept in the dark. Flow cytometry was performed using an 8-color Stratedigm (San Jose, CA) S1000EX apparatus. Data were analyzed using CellCapTure software (Stratedigm).

### 2.4. Cell Proliferation Assay

Cell proliferation assays were conducted using CellTrace™ Violet cell proliferation kit according to the manufacturer's protocol (Invitrogen, Carlsbad, CA). Briefly, cells were incubated in 5 *μ*M of dye in PBS solution for 20 min at room temperature, washed with culture medium, seeded into 6-well plates at 0.4 × 10^6^ cells per well, and cultured for 4 days. Plates were then washed with PBS to remove nonadherent dead cells, and live adherent cells were harvested with 0.25% Trypsin/10 mM EDTA in PBS, stained for intracellular markers, and analyzed by flow cytometry.

### 2.5. Cell Chemoresistance Assay

Cells were seeded into 6-well plate at 0.4 × 10^6^ cells per well, 24 hrs prior to treatment with Vincristine (1 and 10 nM) or Doxorubicin (0.1 and 1 *μ*M) for 48 hrs. For the analysis, plates were washed with PBS to remove dead cells, adherent cells were harvested with Trypsin/EDTA, and viability was determined with Automated Cell Counter NC-200 (Chemometec). After viability evaluation, cells were stained for flow cytometry analysis.

### 2.6. Induction of Myogenic Differentiation

Cells were seeded into 6-well plate at 0.4 × 10^6^ cells per well and after 24 hrs cultured in the presence of 12-O-tetradecanoylphorbol-13-acetate (TPA) (EMD Millipore, Temecula, CA) and GSK126 (AdooQ Bioscience, Irvine, CA), at 0.1 and 5 *μ*M, respectively, as previously described [31]. Cells were harvested at the indicated time points, counted, and stained for intracellular markers.

### 2.7. Statistical Analysis

All statistical analyses were performed using GraphPad Prism 6 (La Jolla, CA) software. One-way ANOVA test with Tukey's correction for multiple comparisons was used to assess the differences among subpopulations in treated and nontreated cell cultures, with *α* set to 0.05. D'Agostino & Pearson normality test had been used beforehand to validate the data normality distribution. In some instances, a paired two-tailed *t*-test has been used.

## 3. Results

### 3.1. Heterogeneity of MYOD1 and Noggin Expression

**Figure 1:**
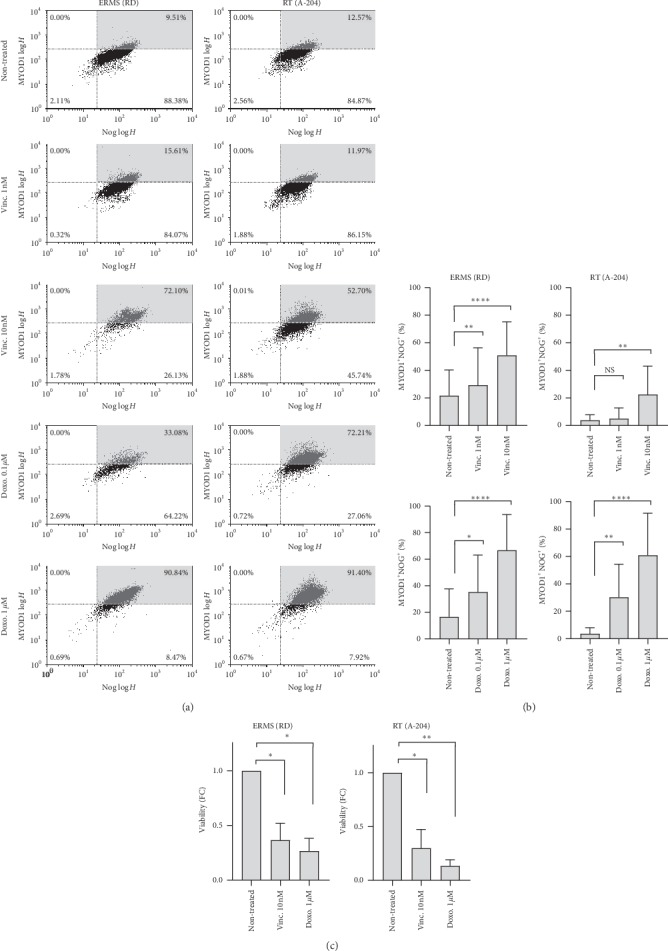
MYOD1+ NOG+ cells predominant survival in RD and A-204 cell cultures after treatment with either Vincristine or Doxorubicin. (a) Plots show examples of flow analysis of RD and A-204 cells treated with Vincristine and Doxorubicin. In grey is highlighted the quadrant representing MYOD1+ NOG+ cells. (b) Percentage of live MYOD1+ NOG+ phenotype cells in RD and A-204 cell cultures after 2 days of treatment either with 1 and 10 nM of Vincristine (top panel) or with 0.1 and 1 *μ*M of Doxorubicin (bottom panel). (c) Absolute numbers of total live cells in RD and A-204 cultures treated with 10 nM of Vincristine or 1 *μ*M of Doxorubicin for 2 days. Data are presented as a ratio of absolute numbers of cells in treated cultures to absolute numbers of cells in nontreated cultures (fold change). Data are shown as mean ± standard deviation (*N* = 11). ^*∗*^*p* < 0.05, ^*∗∗*^*p* < 0.01, ^*∗∗∗*^*p* < 0.001, ANOVA with Tukey's multiple comparison test.

The protein expression of *MYOD1* and *Noggin* genes in RD and A-204 cell lines were evaluated by flow cytometry to assess the distribution of these markers among the cell population. RMS tumors have been reported to be positive for MYOD1 with marked heterogeneity between cells [18], while RT are believed to be negative for MYOD1 [20, 21]. Interestingly, we found MYOD1 to be expressed in approximately 5 to 25% of RD cells and 1 to 10% of A-204 cells, while the majority of cells (≥80%) had MYOD1 below detection levels (Table 1). NOG positive cells (NOG+) always constitute the majority of cells in both RD and A-204 (30 to 90%). Of this population, 10–25% and 1–10% are also positive for MYOD1 (MYOD1+ NOG+) in RD and A-204, respectively. The majority (>90%) of MYOD1+ cells were also positive for NOG while single positive cells (MYOD1+ NOG−) were less than 5% of the MYOD1 population and less than 1% of the total, for both RD and A-204. Importantly, the variability among the observed percentages is high and the populations identified by these 2 markers are in continuum (Figure 1(a)), which strongly suggests dynamic regulation of the expression of the markers and transition of expression profiles among the subpopulations. Further experiments were performed to determine the dynamic of these markers upon chemotherapy and to assess if any of these cell subpopulations may be associated with increased chemoresistance or chemosensitivity.

### 3.2. MYOD1/NOG Expressing Cells Show Higher Levels of Chemoresistance

**Figure 2:**
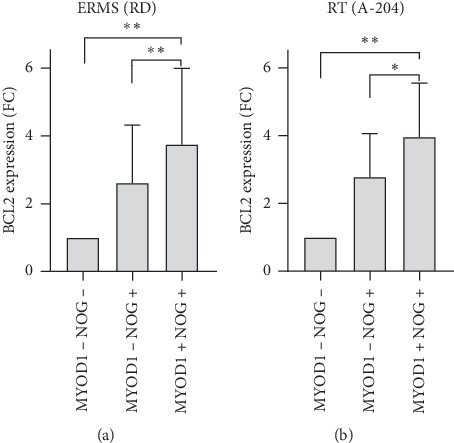
MYOD1+ NOG+ cells express the highest levels of antiapoptotic BCL2 protein among other cells in RD and A-204 cultures. Median fluorescence intensity (MFI) of BCL2 protein in MYOD1/NOG cell subpopulations of RD (left plot) and A-204 (right plot) cell lines. Data are presented as a ratio of BCL2 MFI of MYOD1+ NOG+ cells and MYOD1− NOG+ cells to BCL2 MFI of MYOD1− NOG− cells (fold change), (mean ± standard deviation, *N* = 9). ^*∗*^*p* < 0.05, ^*∗∗*^*p* < 0.01, ANOVA with Tukey's multiple comparison test).

RD and A-204 cells were treated with Vincristine and Doxorubicin, two commonly used chemotherapeutic agents for RMS [32, 33], to examine expression profiles of MYOD1 and NOG markers in the subpopulation of cells surviving acute 48-hour treatment. In both cell lines, the percentage of MYOD1+ NOG+ cells increases in a dose-dependent manner with either treatment (Figures 1(a) and 1(b)). Specifically, at the highest Vincristine treatment dose, the proportion of MYOD1+ NOG+ increased 2.4 times (*p* < 0.001) and 5.9 times (*p* < 0.05) for RD and A-204, respectively (Figure 1(b), top panel). Doxorubicin elicited a similar effect, with an increase of the percentage of MYOD1+ NOG+ of 4.1 times (*p* < 0.001) and 16.6 times (*p* < 0.01) in RD and A-204, respectively (Figure 1(b), bottom panel). Accordingly, a dose-dependent increase in Noggin mRNA levels for both cell lines was observed (Supplementary Figures [Supplementary-material supplementary-material-1] and [Supplementary-material supplementary-material-1]). Interestingly, a dose-dependent increase was observed in MYOD1 mRNA levels in the A-204 cell line ([Supplementary-material supplementary-material-1]), while in RD, mRNA levels were fluctuating resulting in not significantly different means. Currently, there are no cell membrane markers specific for MYOD1/NOG subpopulations analyzed in this study (MYOD1 is a transcription factor and Noggin is a secreted protein) preventing live cells sorting (e.g., by fluorescent activated cell sorting, FACS) for functional analysis. However, we addressed primary resistance associated with each marker by calculating the absolute cell number of each cell subpopulation by flow cytometry in the whole population treated with scalar doses of Vincristine (Supplementary Figures [Supplementary-material supplementary-material-1] and [Supplementary-material supplementary-material-1] and supplementary methods). We observed that IC50 in RD cell line for the whole population was 0.7 nM and for MYOD1− NOG− and MYOD− NOG+ subpopulations was below 0.5 nM, while there was no reduction in the cell number of MYOD1+ NOG+ subpopulation (Supplementary [Supplementary-material supplementary-material-1]). Similar response pattern was observed with A-204 ([Supplementary-material supplementary-material-1]). Doxorubicin at doses above 1uM has high levels of autofluorescence in a wide wavelength range ([34] and personal observation), thus precluding the same dose-response analysis with this drug by flow cytometry. However, the percentages of MYOD1+ NOG+ cells at 0.1 *μ*M and 1 *μ*M Doxorubicin (Figure 1) increased in a dose-dependent manner. Analyzing protein level of BCL2, a well characterized antiapoptotic marker [35, 36], we found higher levels in the MYOD1+ NOG+ subpopulation in untreated RD and A-204 cell lines (Figure 2). In detail, we observed in both cell lines a significant 3.7 and 4.0 times higher BCL2 expression, respectively, in MYOD1+ NOG+ cells when compared to MYOD1− NOG− cells (RD: *p* < 0.01; A-204: *p* < 0.01) and 1.5 and 1.6 times when compared to MYOD1− NOG+ cells (RD:*p* < 0.01; A-204: *p* < 0.05), respectively. Importantly, cells expressing only NOG and not MYOD1 also demonstrated higher BCL2 expression levels than double negative (MYOD1− NOG−) cells, suggesting the involvement of the NOG marker in chemoresistance.

### 3.3. TPA/GSK126 Induces Differentiation of Precursor-Like MYOD1+/NOG+ Cells and Increases Vincristine Efficacy

MYOD1+ NOG+ cells had 2.3 times increased levels of myogenin (MYOG) expression as compared to MYOD1− NOG+ single positive cells (*p* < 0.001), and no MYOG was detected in MYOD− NOG− double negative cells. MYOG is one of the myogenic regulatory factors that drive skeletal muscle cell differentiation [37, 38]. MYOG expression in cells follows and overlaps with the expression of MYOD1 in a tight temporal manner at later stages of myogenesis, and it has an indispensable role in terminal differentiation of myoblasts [39]. Importantly, MYOD1+ NOG+ cells had 2.0 times higher level of inhibitor of differentiation 1 (ID1) expression (ID1 mean fluorescence intensity) as compared to MYOD1− NOG+ single positive cells (*p* < 0.01) as assessed by flow cytometry.

**Figure 3:**
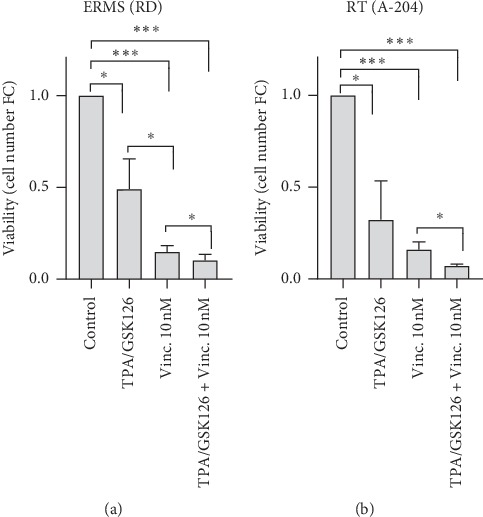
Differentiation therapy increases the effectiveness of chemotherapy. Absolute numbers of total live cells RD (a) and A-204 (b) in cultures treated for 6 days with TPA/GSK126, 10 nM of Vincristine, or combination of Vincristine and TPA/GSK126. Data are presented as a ratio of absolute numbers of cells in treated cultures to absolute numbers of cells in nontreated cultures (fold change) (mean ± standard deviation, *N* = 4). ^*∗*^*p* < 0.05, ^*∗∗∗*^*p* < 0.001, ANOVA with Tukey's multiple comparison test for the analysis between the groups.

Since resistant-to-chemotherapy MYOD1+ NOG+ cells demonstrated features of undifferentiated cells and their percentage increased upon chemotherapy, a combined-treatment approach of Vincristine and 12-O-tetradecanoylphorbol-13-acetate (TPA)/GSK126 (TPA/GSK126) was tested. TPA/GSK126 has been shown to induce differentiation of RMS cell lines [31] but has not yet been tested in combination with chemotherapy. After a 6-day TPA/GSK126 treatment, there was a statistically significant increase in the level of MYOG protein in MYOD1+ NOG+ cells, indicating induction of differentiation. Specifically, in RD cell line, MYOG expression increased 1.2 times in TPA/GSK126-treated MYOD1+ NOG+ cells as compared to untreated MYOD1+ NOG+ cells (*p* < 0.001), and in A-204 cell line 1.3 times, correspondingly (*p* < 0.01). Although the average upregulation of MYOG expression was modest, at about 20% increase, the dispersion of MYOG protein levels among cell subpopulations in RD was strongly reduced in MYOD1+ NOG+ cells upon treatment, indicating a robust induction of its expression in cells with low levels of MYOG (up to 2 folds). TPA/GSK126 treatment not only induced expression of MYOG suggesting cell differentiation but also exhibited a therapeutic effect by reducing on average 2-fold absolute numbers of live cells in RMS cell cultures (*p* < 0.001) (Figure 3). Importantly, when administered in combination with Vincristine, an increased efficacy by further reduction of cell viability was observed. Specifically, in RD Vincristine alone decreased the number of live cells 6.7 times, as compared to nontreated cells, TPA/GSK126 2 times, and the combination of Vincristine with TPA/GSK126 9.7 times, correspondingly (Figure 3). In RD the combination of Vincristine and TPA/GSK126 was therefore 1.5 times more effective than Vincristine alone and 4.8 times more effective than TPA/GSK126 alone. In A-204 cell cultures Vincristine decreased the number of live cells 6.2 times as compared to nontreated cells, TPA/GSK126 3.1 times, and the combination of Vincristine with TPA/GSK126 14.2 times, a 2.3-time increase in effectiveness of Vincristine and 4.6-time increase in effectiveness of TPA/GSK126.

## 4. Discussion

Treatment of RMS with standard chemotherapeutic agents often fails due to onset of resistance and recurrence. Since RMS tumor cells demonstrate an intratumor differential response to these agents, a degree of heterogeneity among tumor cells can be postulated. In this in vitro study, Vincristine and Doxorubicin chemoresistant subpopulations were identified as cells expressing the markers MYOD1 and NOG in two patient-derived soft-tissue tumor models (A-204, a rhabdoid tumor cell line, and RD, an embryonal rhabdomyosarcoma cell line). MYOD1+ NOG+ cell subpopulation demonstrated increased levels of BCL2 protein expression prior to treatment, thus indicating that resistance to cell death is an intrinsic property of this cell subpopulation, resulting in primary resistance to chemotherapeutic agents.

RMS and RT cells aberrantly proliferate and retain undifferentiated features. Nonetheless, within the tumor, cells may have different degrees of differentiation. The dynamic expression of MYOD1/NOG markers suggests that these cells may behave as early progenitors able to sustain tumor growth. MYOG is one of the myogenic regulatory factors that is downstream of MEF2, MYF5, MRF4, and MYOD1 that drive skeletal muscle cell differentiation [37, 38]. MYOG expression in cells follows and overlaps with the expression of MYOD1 in a tight temporal manner at later stages of myogenesis and plays an indispensable role in terminal differentiation of myoblasts [39]. Additionally, these results demonstrated that MYOD1/NOG double positive cells are less differentiated than the rest of the cells. Since RMS is considered to be a result of failure of precursor cells to complete the differentiation process to skeletal muscle, the possibility of using differentiation therapy to address the problem of MYOD1/NOG chemoresistant cells was investigated. The concept of differentiation therapy was proposed as early as the 1970s based on the idea that the malignant cells (perhaps Cancer Stem Cells or Tumor Propagating Cells) could differentiate into less aggressive or benign cells, allowing differentiation to be an alternative or complementary strategy to chemotherapy-mediated cytotoxicity [40–42]. It has been shown that differentiation therapy can reduce malignancy by preventing tissue invasion and metastasis [43]. Leukemia is by far the most studied cancer for differentiation therapy and the only cancer with a successful application [44]. However, with solid tumors this strategy has shown limited results [45, 46] and it has therefore been proposed as a potential adjuvant to other therapies. A few studies have evaluated a differentiation therapy strategy in various sarcomas [47–51], but none have evaluated such approach in combination with standard chemotherapy and its effects on chemoresistant cells. Increased ID1 protein expression is characteristic of precursor cells [25, 52]. Altogether this data suggest that MYOD1+ NOG+ cells are cells halted in the process of differentiation, expressing high levels of MYOG and ID1.

Since RD and A-204 MYOD1+ NOG+ cells (1) demonstrate resistance to chemotherapy and (2) show features of undifferentiated cells and (3) their percentage increases upon chemotherapy, Vincristine was tested in combination with 12-O-tetradecanoylphorbol-13-acetate (TPA) and GSK126 (TPA/GSK126). TPA/GSK126 has been shown to induce differentiation of RMS cell lines [31] but has not yet been tested in combination with chemotherapy. TPA promotes growth arrest and skeletal muscle differentiation in RD [53] via PKC*α* activation [54]. GSK126 is a selective inhibitor of EZH2, a histone methyltransferase that epigenetically suppresses the transcription of myogenic genes [48]. Here, in addition to the direct effect of the differentiating agent TPA/GSK126, the effect of their combination with chemotherapy on a chemoresistant cell population was examined. The differentiating agent TPA/GSK126 induced expression of the differentiation marker MYOG in RD and A-204 cells. When in combination with Vincristine, increased cell death compared to Vincristine or TPA/GSK126 alone was demonstrated.

The mechanism(s) by which expression of MYOD1 and Noggin affects chemoresistance requires further elucidation. MYOD1 and NOG might not be actively involved in the process, but could be indicators of, or associated with, other more fundamental mechanisms. The coexpression and positive correlation of MYOD1, NOG, and BCL2 expression levels warrant further mechanistic investigations.

## 5. Conclusions

In this study, the myogenic determination factor 1 (MYOD1) and the morphogenetic protein Noggin (NOG) were investigated in an embryonal rhabdomyosarcoma (ERMS) and a rhabdoid tumor (RT) cell line. Advanced, recurrent, and/or metastatic RMS and RT exhibit poor response to treatment. One of the main mechanisms behind resistance to treatment is believed to be intratumoral heterogeneity. Heterogeneous expression levels of MYOD1 and NOG were observed in both cell lines, with the MYOD1+ NOG+ subpopulation of cells crucially showing primary resistance to Vincristine and Doxorubicin, two commonly used chemotherapies for ERMS and RT. In addition to the expression of the antiapoptotic marker BCL2 indicating chemoresistance, MYOD1+ NOG+ cells expressed markers of undifferentiated cells such as ID1 and MYOG. Subsequent testing of a 3-drug combination of Vincristine with the TPA/GSK126 differentiation therapy approach demonstrated a partial override of cells chemoresistance.

## Figures and Tables

**Table 1:** Percentage of MYOD1/NOG cell subpopulations in RD and A-204 cell lines.

	MYOD1− NOG−	MYOD1+ NOG−	MYOD1− NOG+	MYOD1+ NOG+
RD (*n* = 13)	29.3 ± 36.4	0.5 ± 0.9	59.8 ± 36.2	10.4 ± 13.8
A-204 (*n* = 13)	27.2 ± 35.6	0.2 ± 0.4	69.3 ± 35.6	3.4 ± 4.2

Cells were harvested for the analysis when cell line cultures reached 70–80% confluence. Data are presented as mean ± standard deviation. *n*: the number of independent observations.

## Data Availability

All flow cytometry data, cell counts, and viability data used to support the findings of this study are available from the corresponding author upon request.

## Supplementary Materials

Supplementary Materials Figure S1: relative MYOD1 and Noggin mRNA expression levels in treated ERMS and RT cell lines. Figure S2: dose-response curves of ERMS RD cell line to Vincristine treatment. Figure S3: dose-response curves of RT A-204 cell line to Vincristine treatment.
