# Supplementary material for: Induction of Myogenic Differentiation Improves Chemosensitivity of Chemoresistant Cells in Soft-Tissue Sarcoma Cell Lines
Source: Sarcoma. 2020 Mar 26;2020:8647981. doi: 10.1155/2020/8647981 (PMC7136814; doi:10.1155/2020/8647981)
Supplement: Supplementary Materials — Figure S1: relative MYOD1 and Noggin mRNA expression levels in treated ERMS and RT cell lines. Figure S2: dose-response curves of ERMS RD cell line to Vincristine treatment. Figure S3: dose-response curves of RT A-204 cell line to Vincristine treatment. [file 8647981.f1.pdf]

Supplementary Materials

Figure S1

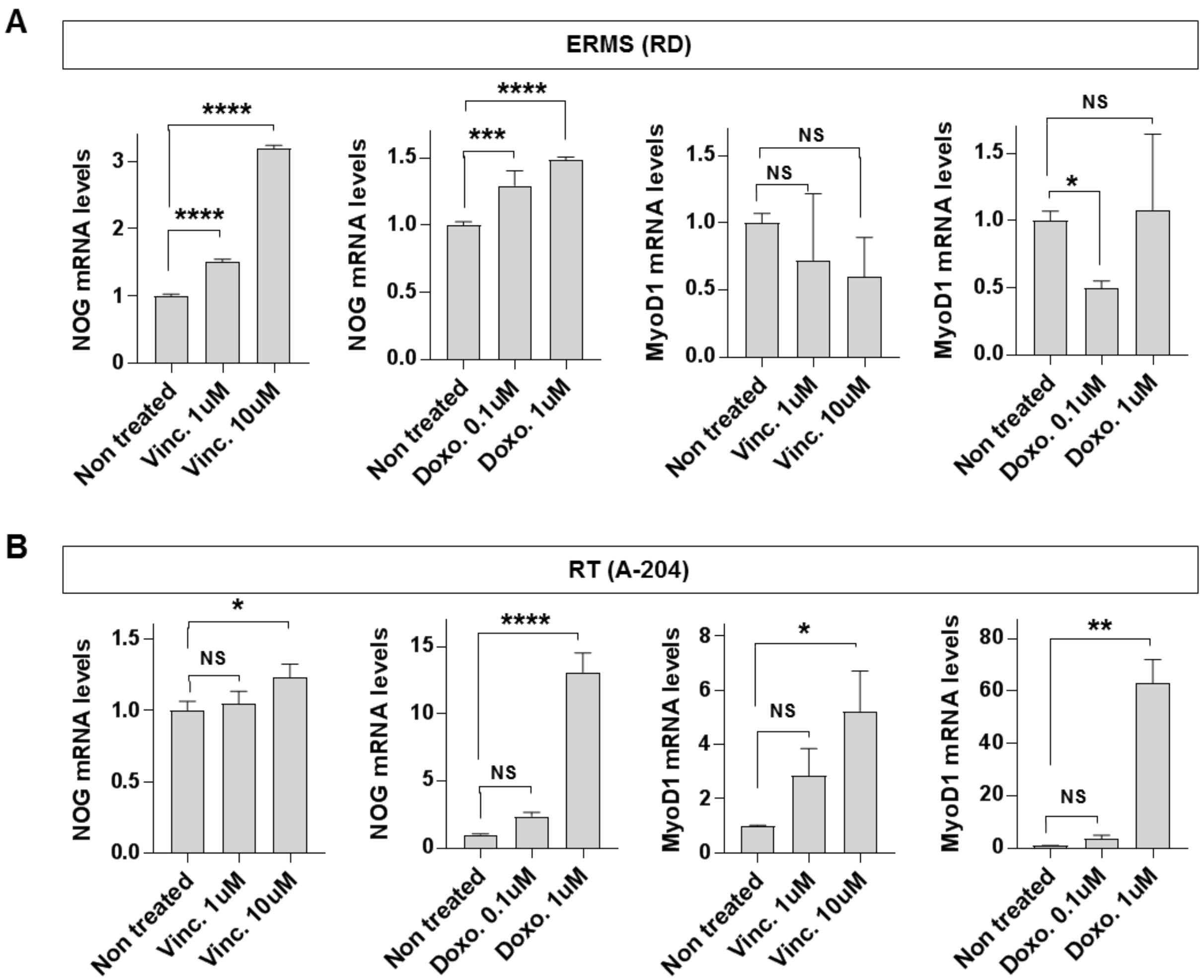

Figure S2

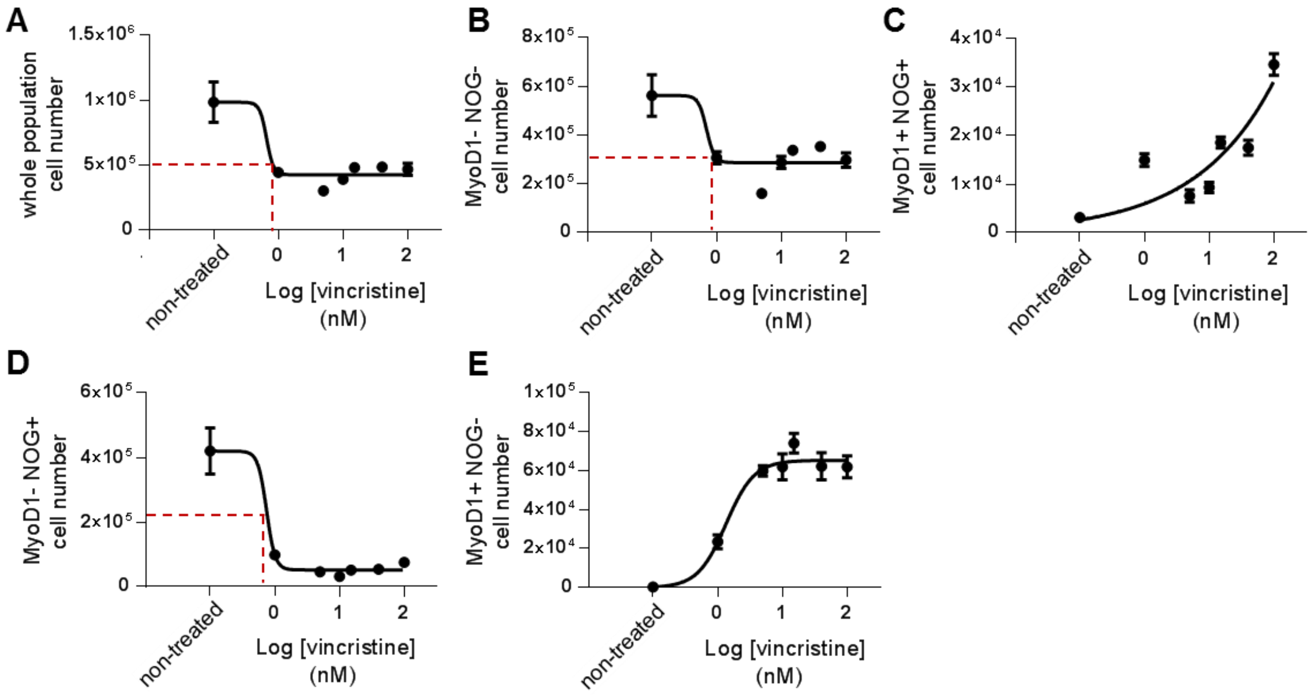
A
whole population cell number
1.5x10^6
1x10^6
5x10^5
0
non-treated
0
1
2
Log [vincristine] (nM)
B
MyoD1- NOG- cell number
8x10^5
6x10^5
4x10^5
2x10^5
0
non-treated
0
1
2
Log [vincristine] (nM)
C
MyoD1+ NOG+ cell number
4x10^4
3x10^4
2x10^4
1x10^4
0
non-treated
0
1
2
Log [vincristine] (nM)
D
MyoD1- NOG+ cell number
6x10^5
4x10^5
2x10^5
0
non-treated
0
1
2
Log [vincristine] (nM)
E
MyoD1+ NOG- cell number
1x10^5
8x10^4
6x10^4
4x10^4
2x10^4
0
non-treated
0
1
2
Log [vincristine] (nM)

Figure S3

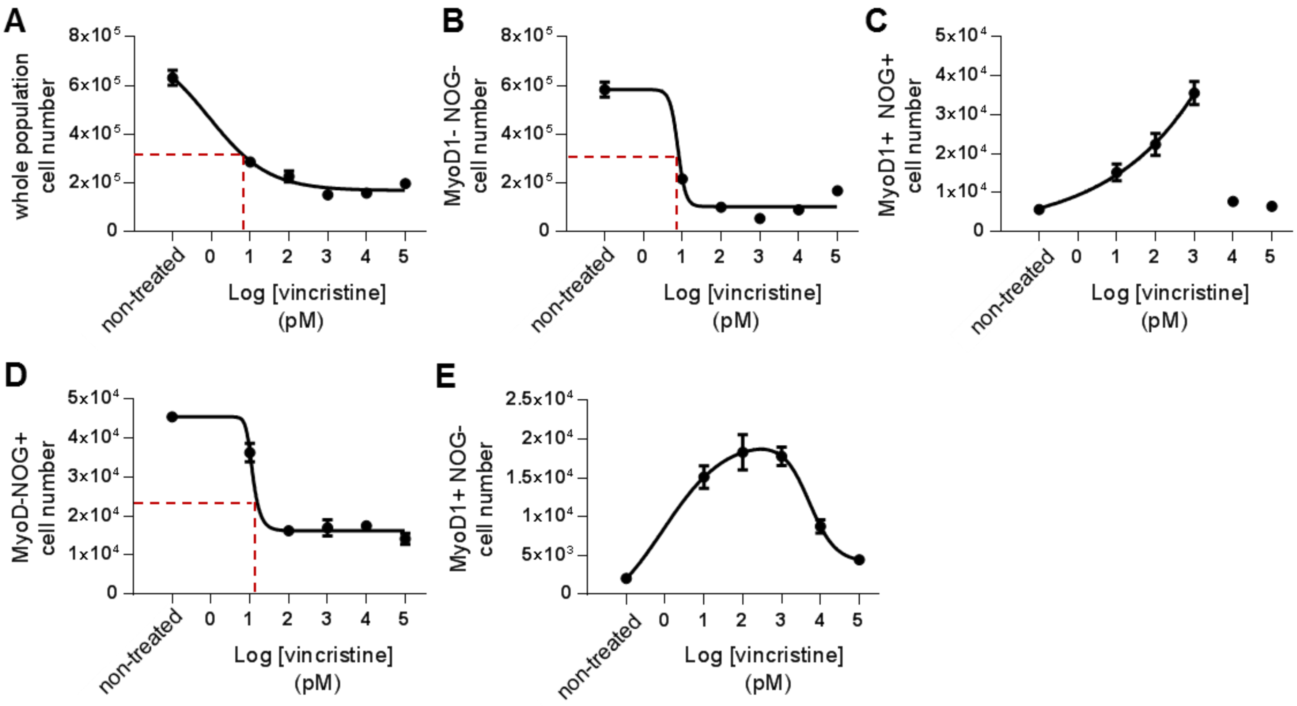
A
whole population cell number
B
MyoD1- NOG- cell number
C
MyoD1+ NOG+ cell number
D
MyoD-NOG+ cell number
E
MyoD1+ NOG- cell number
non-treated
Log [vincristine] (pM)

Supplementary figures legends

Figure S1. Relative MyoD1 and Noggin mRNA expression levels in treated ERMS and RT cell lines. Histograms show relative expression levels of Noggin (NOG) and MyoD1 normalized to non-treated control in **(A)** ERMS cell line RD and in **(B)** RT cell line A-204 (Mean ± standard deviation, N=3). *$*p<0.05$ $**p<0.01$ $***p<0.001$ $****p<0.0001$,* ANOVA with Tukey's multiple comparisons test).

Figure S2. Dose reponse curves of ERMS RD cell line to vincristine treatment. Drug treatment dose responses for **(A)** whole cell population, R^2=0.85, IC_{50}=0.7 nM **(B)** double negative (MyoD1- NOG-), R^2=0.8, IC_{50}=0.5 nM **(C)** double positive (MyoD1+ NOG+), R^2=0.76 **(D)** MyoD1- NOG+ (single positive) subpopulation, R^2=0.85, IC_{50}=0.4 nM **(E)** MyoD1+ NOG- (single positive) subpopulation, R^2=0.95. Plots represent viable cell numbers versus drug concentrations transformed to logarithmic scale. Best fit curves (Mean ± Standard Deviation, N=3) are shown. Non-linear regression using least squares (ordinary) fit was applied for (A, B, D, E), and exponential growth using least squares (ordinary) fit for (C). Red dotted lines highlight the calculated IC_{50}.

Figure S3. Dose reponse curves of RT A-204 cell line to vincristine treatment. Drug treatment dose responses for **(A)** whole cell population, R^2=0.98, IC_{50}=0.70 pM **(B)** double negative (MyoD1- NOG-), R^2=0.96, IC_{50}=0.8 pM **(C)** double positive (MyoD1+ NOG+), R^2=0.97, **(D)** MyoD1-, NOG+ (single positive) subpopulation, R^2=0.86, IC_{50}=17.7pM **(E)** MyoD1+ NOG- (single positive) subpopulation, R^2=0.97. Plots represent viable cell numbers versus drug concentrations transformed to logarithmic scale. Best fit curves for 3 replicate experiments (Mean ± Standard Deviation) are shown. Non-linear regression dose-response (four parameters) using least squares (ordinary) fit was applied for (A, B, D), exponential growth using least squares (ordinary) fit for (C), and non-linear regression bell-shaped dose-response using least squares (ordinary) fit was applied for (E). Red dotted lines highlight the calculated IC_{50}.

Supplementary methods

Real time qRT-PCR

Total RNA was extracted using RNAeasy Plus Mini Kit (Qiagen) according to the manufacturer's instructions. Optional on-column DNA digestion step was performed with RNase-Free DNase according to the manufacturer's instructions (Qiagen). Reverse transcription was performed with SuperScript IV First Strand Synthesis (Invitrogen). Real-time RT-PCR was performed with a SYBR green master Mix kit (Applied Biosystems). Relative mRNA levels were normalized to HPRT and calculated by $\Delta\Delta$CT method.

The primers are listed below:

MYOD1 (F: 5'-TGCTGGACAGGCAGTCTA-3', R: 5'-CTCCGACGGCATGATGG-3')

NOG (F:5'-GCAGCGTCTCGTTCAGAT-3', R: 5'-CCAGCACTATCTCCACATCC-3')

HPRT (F: 5'-GCGATGTCAATAGGACTCCAG-3', R:5'-TTGTTGTAGGATATGCCC-TTG A-3'

Drug dose-response curves

Cell lines were seeded at 0.3 x 106 cells per well and treated with drug for 48 hours. RD cells were treated with 1nM, 5nM, 10nM, 15nM, 40nM and 100nM of vincristine, A-204 cells - with 0.01nM, 0.1nM, 1nM, 10nM and 100nM of vincristine. Cells were collected at 48 hours after twice washing wells with PBS to remove dead cells. Cells absolute numbers and viability were obtained using Chemometec NC-200. Then cells were immediately processed for intracellular flow cytometry staining as described in the corresponding Material and Methods section. Percentages of each cell subpopulation (MyoD1- NOG-, MyoD1+ NOG+, MyoD1- NOG+ and MyoD1+ NOG-) was obtained by flow cytometry analysis and absolute numbers of these cell subpopulations were calculated.

57
